# English- and Spanish-speaking U.S. adults’ perceptions of the most common reasons for abortion: a study of open-ended data before and after *Dobbs v. Jackson*

**DOI:** 10.1186/s12978-025-02039-5

**Published:** 2025-07-18

**Authors:** Lucrecia Mena-Meléndez, Xiana Bueno, Kyla M. Cary, Nana Amma Asamoah, Brandon L. Crawford, Ronna C. Turner, Kristen N. Jozkowski

**Affiliations:** 1 Department of Applied Health Science, Indiana University, 1033 E 3rd St, Bloomington, IN 47405 USA; 2 Educational Statistics and Research Methods, University of Arkansas, 100 Graduate Education Building, Fayetteville, AR 72701 USA; 3 Kinsey Institute for Research in Sex, Gender, and Reproduction, Indiana University, Bloomington, IN 47405 USA

**Keywords:** Abortion attitudes, Abortion reasons, Open-ended data, Multi-language research, Longitudinal data, Dobbs decision

## Abstract

**Background:**

The 2022 *Dobbs v. Jackson* decision, which overturned *Roe v. Wade*, has given individual states more capacity to legislate abortion. State legislators have and continue to design and pass laws that restrict or ban abortion, often naming exceptions based on specific reasons (i.e., fetal health, woman’s health, rape). Given that these reasons often do not align with those reported by abortion-seekers, it is crucial to assess whether the U.S. public accurately understands why people seek abortions. This study explored a sample of U.S. adults’ perceptions of the three most common reasons *why* someone might get an abortion.

**Methods:**

We analyzed open-ended data from two waves of a 2022 longitudinal survey (*n* = 681 participants; *n* = 2,043 responses per wave; *n* = 4,086 total responses) collected before and after the *Dobbs* decision in English and Spanish via Ipsos’s KnowledgePanel®. We explored three main research questions: (1) What does the U.S. public perceive to be the most common reasons for someone to seek abortion? (2) Are there differences in perceived reasons before and after the *Dobbs v. Jackson* decision? (3) Are there differences in perceived reasons across languages?

**Results:**

Findings indicated that people perceive the three most common reasons to seek abortion to be: *unwanted/unplanned pregnancy reasons*, *violence-related reasons*, and *health reasons*. After the *Dobbs* decision, there was an increase in respondents mentioning that people have abortions for *health reasons* and *financial reasons*, and a decrease in responses related to *unwanted/unplanned pregnancy reasons, not ready/unprepared reasons,* and *partner-related reasons*. Additionally, we found significant differences in perceptions between languages (i.e., English and Spanish). We also note discrepancies between perceived reasons among our sample and reasons reported by abortion patients in national studies.

**Conclusions:**

This study underscores the public’s misconceptions of reasons for seeking abortion and the importance of correcting such misunderstandings to ensure alignment of public sentiment and legislative and judicial policy post-*Dobbs*.

## Introduction

The reasons *why* people have abortions are numerous. While the general public might create subjective perceptions of why someone would seek abortion [1], only people who have had abortions themselves know the reasons behind their decisions. For almost five decades in the United States (U.S.), abortion was a constitutional right up to fetal viability through *Roe v. Wade* [2]. During this period, the reasons *why* people had abortions were considered a private matter, and thus, public perceptions of those reasons were, in practice, irrelevant. However, the U.S. Supreme Court’s 2022 *Dobbs v. Jackson* decision overturned *Roe v. Wade* and gave individual states more capacity to legislate abortion than they were previously able to because of *Roe v. Wade*. This has potentially changed the relevance of people’s perceptions of reasons for seeking and obtaining abortion, as legislators continue to design and pass laws that restrict or ban abortion, often naming exceptions based on specific reasons (i.e., fetal health, woman’s health, rape).^1^ However, these reasons often do not align with the reasons that are most frequently reported by abortion-seekers [5, 6]. Abortion access and policy continue to vary widely by state, with some states enacting total bans and others maintaining or expanding access. These differences are documented in projects like #WeCount, which tracks state-level changes in abortion provision and highlights how legal restrictions may shape both access and public perceptions across state lines [7]. Moreover, since 2022, some states have proposed actions that have put abortion legislation directly in the hands of voters through ballot initiatives [8]. Thus, it is imperative to assess whether the U.S. public has an accurate perception of *why* people have abortions.

Circumstances motivating an abortion, or reasons behind an abortion decision, influence the public’s attitude toward the legality of abortion. Since the 1970s, the General Social Survey (GSS) has asked the U.S. public whether it should be possible for a pregnant woman to obtain a *legal* abortion in six different circumstances: (1) “if there is a strong chance of serious defect in the baby,” (2) “if she is married and does not want any more children,” (3) “if the woman’s own health is seriously endangered by the pregnancy,” (4) “if the family has a very low income and cannot afford any more children,” (5) “if she became pregnant as a result of rape,” and (6) “if she is not married and does not want to marry the man.” There is also a seventh item—“if she wants it [an abortion] for any reason.” Abortions performed for health-related reasons (e.g., fetal defect, life endangerment) have often garnered greater endorsement of legal abortion among U.S. adults compared with those sought for socioeconomic-related reasons, such as being low-income or unmarried [9]. Yet, whether these circumstances continue to reflect perceived and actual reasons for abortion today remains uncertain. To address this gap, we examined U.S. adults’ perceptions of the three most common reasons *why* someone would seek abortion. Given the magnitude of the *Dobbs v. Jackson* decision, we also explored differences in perceptions before and after the decision. Finally, because our sample comprised people who completed the survey in both English and Spanish, we also examined differences in perceptions between languages in an exploratory fashion.

### Reasons for abortion reported by women who have had abortions in the U.S

Across several decades, scholars have directly assessed reasons and circumstances for abortion as provided by pregnant people. Historically, the most frequently reported reasons for seeking abortion were concerns that having a baby would change their life (78%), not being able to afford a child (69%), not wanting to be a single parent, or relationship problems (52%), and not being ready for the responsibility of raising a child (36%) [10]. Almost two decades later, Finer et al. [11] found a nearly identical pattern of the most frequently reported reasons for abortion among abortion patients: (1) dramatic life changes (74%), (2) financial reasons (73%), and (3) partner-related reasons (48%). In contrast, public discourse and legislation often discuss the fetus’ or woman’s health as common reasons for abortion [12, 13]. However, only 14% and 8% [10] and 13% and 12% [11] of abortion patients, respectively, reported fetal health or woman’s health as reasons for seeking abortion. The most recently gathered data on abortion reasons from the Turnaway Study [5] also revealed the following most frequently cited reasons for seeking abortion: (1) financial reasons (40%), (2) reasons related to timing (36%), (3) partner-related reasons (31%), and (4) need to focus on other children (29%). In their literature review of multiple studies, Kirkman et al. [6] identified three common groups of reported reasons: (1) women-focused reasons (e.g., woman’s health, wrong timing, do not want (more) children), (2) other-focused reasons (e.g., partner, child concerns, influence from others), and (3) material reasons (e.g., financial and housing limitations, unreadiness). Collectively, these findings suggest there are myriad reasons people seek abortion, with notable consistency over time.

It is also important to highlight that people often report multiple reasons for seeking abortion. In 1988, a mere 7% of abortion patients reported just one reason for seeking abortion [10]. Kirkman et al. [6] similarly identified that women’s reasons for seeking abortion were numerous and complex. In qualitative interviews, women reported reasons for abortion that are a product of “multiple dimensions of complicated life situations” [11] and explained that it is difficult to narrow down the *main* reason for having an abortion when reporting multiple reasons [5]. Thus, there are complexities embedded in the abortion-seeking decision-making process, and perceived reasons *why* someone may seek or have an abortion should be assessed more comprehensively.

### Public perceptions of the reasons people seek abortion in the U.S

Although decades of research have explored pregnant people’s reasons for seeking abortion, less research has considered the public’s perceptions of *why* someone may seek abortion. To assess perceptions, scholars have underscored the importance of distinguishing between studies that either allow respondents to report perceived reasons through open-ended formats or through qualitative interviews from studies that provide respondents with a list of possible researcher-generated reasons [6]. One of the few studies that employed the former method explored perceived reasons for seeking abortion shared in nationally representative interview data. Bruce et al. [1] identified four main themes that summarized people’s “abortion imaginary” or perceptions of *who* seeks abortion and *why*: (1) economic decision-making, (2) relationship precarity, (3) emotional fragility, and (4) maternal inevitability (i.e., the assumption that all women will eventually become mothers). Findings revealed that people’s perceptions of the type of women who seek abortion (i.e., young, White, unpartnered, childless women without economic stability) did not necessarily match the reality of most abortion-seekers (i.e., Black/Latina women in their 20 s who already have children). Thus, these perceptions may be driven by stereotypes or misconceptions. In contrast, participants who drew upon “abortion exemplars” (i.e., someone who had an abortion, whether themselves or someone they knew) were less likely to draw upon a stereotyped other.

Alternatively, surveys, such as the GSS, have employed researcher-generated reasons to assess the public’s attitudes toward *legal* abortion using the six aforementioned circumstances. It is possible that people interpret the circumstances asked in survey items as common reasons why people seek abortion. It is also possible that because these circumstances are asked commonly in national polls and surveys [14], they may be more salient circumstances that come to people’s minds when thinking about abortion [15]. However, despite decades of surveys relying on these items to assess views on abortion legality, the reasons provided by abortion patients do not align with some of the reasons provided in most close-ended survey items. For example, abortion patients’ most frequently mentioned reasons for seeking abortion, related to concerns about life changes [11] and limiting childbearing [5, 16, 17], are not well-reflected in the GSS circumstances. Additionally, socioeconomic concerns and partner-related issues are narrowly defined in GSS circumstances as “if the family has a very low income and cannot afford any more children” and “if she is not married and does not want to marry the man.” Importantly, the GSS does not assess whether these reasons are indeed common. Rather, these items assess people’s attitudes toward legal abortion given circumstantial factors, and research assessing people’s actual perceptions of the reasons why abortion is sought is quite limited.

### Current study

We sought to build upon and extend the small body of literature that explores the public’s perceptions of the most common reasons people seek abortion. Additionally, we consider whether the reasons provided by the public align with the actual reasons reported by abortion-seekers and the reasons included in surveys. This study uses open-ended data from a 2022 web-based longitudinal survey to explore a sample of U.S. adults’ perceptions of the three most common reasons *why* someone gets an abortion. We pursued three specific research questions: (RQ1) What does the U.S. public perceive to be the most common reasons for someone to seek abortion? (RQ2) Are there differences in perceived reasons before and after the *Dobbs v. Jackson* decision? (RQ3) Are there differences in perceived reasons across languages (i.e., English and Spanish)?

## Methods

### Study design and data collection

In 2022, we launched an online longitudinal survey using Ipsos’s probability-based KnowledgePanel®, a web-based panel designed to be representative of the U.S. household population [18]. Panel members are randomly selected using address-based sampling methods, specifically from the U.S. Postal Service’s Delivery Sequence File, which covers nearly 100% of U.S. households. This approach ensures that the panel includes hard-to-reach individuals, such as those without internet access, young adults, and minority subgroups. Households without internet connectivity are provided with web-enabled devices and internet service to facilitate their participation. Upon recruitment, panelists complete an initial demographic survey, allowing for efficient sampling and weighting in subsequent studies. Surveys for the present study were self-administered online, ensuring comprehensive coverage and representation of the U.S. adult population in survey research. Eligibility criteria for participation in our study included being 18 years of age or older and residing in the United States.

The purpose of the study was to assess U.S. adults’ awareness, knowledge, and attitudes regarding abortion before and after the *Dobbs v. Jackson* decision announcement.^2^ We launched Wave 1 of the survey in June 2022 (*n* = 1,014) before the decision announcement. As the announcement date was unknown, the survey was in the field between June 9 and June 26, 2022, with most of the sample (93.2%) completing the survey before the *Dobbs* announcement on June 24 th at 10:00 am EST. Wave 2 of the survey was fielded with the same participants from Wave 1 between October 17 and November 11, 2022 (*n* = 792), several months after the *Dobbs* decision was issued and many state-level abortion laws had changed. This timing was selected to balance two key considerations: allowing sufficient time after the *Dobbs* decision for newly enacted abortion laws to take effect and for media coverage to reach the broader public, while still ensuring that public perceptions were assessed within a relevant window of public attention. Additionally, the timing coincided with the 2022 U.S. midterm elections, during which abortion-related issues were a significant part of political discourse, potentially influencing public perceptions. Our aim was not to capture heightened or transient reactions immediately after *Dobbs*, but rather to assess more stabilized public perceptions after initial legal and media responses had occurred. The attrition rate was 21.9%. To increase the validity of our research questions and design, we excluded participants (*n* = 58) who completed Wave 1 after June 24, 2022, at 10:00 am EST—the day and time when the decision was announced. We administered our survey in English and Spanish, with approximately 87.1% of respondents answering in English (*n* = 593) and 12.9% of respondents answering in Spanish (*n* = 88). The multi-language design of the survey items followed a parallel translation development approach that adhered to the Translate, Review, Adjudicate, Pretest, and Document (TRAPD) framework [19]. This approach entailed a team-based translation process with several iterations of review and reconciliation. Modifications to the source (i.e., English) items were made when a more adequate translation from the target language (i.e., Spanish) was required. The Institutional Review Board at Indiana University approved all materials and study protocols for both waves before data collection, in accordance with the ethical principles of the Belmont Report and the U.S. Common Rule (45 CFR 46). We obtained informed consent from all participants before beginning each survey. Participants received an incentive through Ipsos’ KnowledgePanel® administration points system. Most respondents earn approximately 1,000 points per completed survey, equivalent to $1 USD, with additional points provided for longer surveys [20]. Participants were 345 (50.66%) women and 336 (49.34%) men,^3^ with a mean age of 51.71 (*SD* = 17.00). Additional sample demographic characteristics can be found in Table 1. In terms of language, we assessed whether Spanish-language respondents were monolingual or bilingual using one language-related measure collected by Ipsos as part of their standard off-panel demographic profile, which classifies Hispanic respondents as English proficient, bilingual, or Spanish proficient based on English-speaking ability. The Ipsos measure is based on a follow-up question asked of Hispanic panelists who report speaking a language other than English at home (*n* = 144). These respondents are asked to rate how well they speak English (*very well*, *well*, *not well*, *not at all*), and based on their response, Ipsos categorizes them as English proficient, Bilingual, or Spanish proficient. Table 2 presents the distribution of respondents’ language proficiency. Findings revealed that Spanish-language respondents were a mix of monolingual and bilingual individuals. For additional context of language proficiency, we also include Table 6 in the Appendix, which presents results from participants’ self-rated ability in English and Spanish across six everyday domains—speaking, reading, understanding television, understanding radio, writing, and understanding music—adapted from a modified version of the Bidimensional Acculturation Scale for Hispanics (BASH). In this study, we analyzed the following open-ended survey item: “*What do you think are the three most common reasons someone gets an abortion?*” (in Spanish, “*¿Cuáles cree que son las tres razones más comunes por las que alguien tiene un aborto intencional?*”). Participants were provided with three text boxes to enter their answers for reason 1, reason 2, and reason 3. This item was replicated in Wave 1 and Wave 2 of the survey, which resulted in the possibility of six total responses per participant. Our analytical sample comprises all respondents who responded to at least one of the three reasons in both waves. Thus, we excluded *n* = 111 participants (52 participants skipped the question in both waves, 40 skipped in Wave 1 only, and 19 skipped in Wave 2 only). Our final analytical sample comprised 681 participants who could have provided up to three reasons for getting an abortion for a total of 2,043 possible reasons for each wave of the survey, and thus, 4,086 total possible responses. Of all 4,086 possible responses, 105 were left blank, 33 were determined to be uncodable, 79 did not contain enough context to make a coding determination, and 18 indicated “don’t know.”

**Table 1:** Demographic characteristics of the analytic sample (*N* = 681)

	**Pre-*Dobbs*** ***n*/Mean (%/SD)**	**Post-*Dobbs*** ***n*/Mean (%/SD)**
Sex		
Male	336 (49.34)	336 (49.34)
Female	345 (50.66)	345 (50.66)
Age group		
18–34	131 (19.24)	131 (19.24)
35–49	176 (25.84)	176 (25.84)
50–64	182 (26.73)	182 (26.73)
65 +	192 (28.19)	192 (28.19)
Mean age	51.71 (17.00)	51.71 (17.00)
Race/ethnicity		
White	433 (63.58)	433 (63.58)
Black or African American	62 (9.10)	62 (9.10)
Other	25 (3.67)	25 (3.67)
Hispanic or Latinx	144 (21.15)	144 (21.15)
2 + races	17 (2.50)	17 (2.50)
Education		
No high school diploma	61 (8.96)	61 (8.96)
High school graduate	175 (25.70)	175 (25.70)
Some college or associate degree	164 (24.08)	164 (24.08)
Bachelor’s degree	152 (22.32)	152 (22.32)
Master’s degree or more	129 (18.94)	129 (18.94)
Political party		
Republican	–	169 (24.93)
Democrat	–	224 (33.04)
Independent	–	232 (34.22)
Something else	–	53 (7.82)
Religion		
Evangelical Christian	117 (17.31)	120 (17.88)
Mainline Christian/Protestant	111 (16.42)	111 (16.54)
Catholic	183 (27.07)	182 (27.12)
Mormon	12 (1.78)	9 (1.34)
Orthodox Christian	3 (0.44)	4 (0.60)
Jewish	23 (3.40)	22 (3.28)
Muslim	2 (0.30)	2 (0.30)
Buddhist	5 (0.74)	10 (1.49)
Hindu	1 (0.15)	1 (0.15)
Atheist	60 (8.88)	51 (7.60)
Agnostic	78 (11.54)	73 (10.88)
Other	81 (11.98)	86 (12.82)
Abortion identity^a^		
Pro-choice	334 (49.26)	346 (51.11)
Pro-life	201 (29.65)	191 (28.21)
Equally pro-choice and pro-life	59 (8.70)	71 (10.49)
Neither pro-life nor pro-choice	42 (6.19)	30 (4.43)
Prefer not to answer	42 (6.19)	39 (5.76)
Area of residence		
Urban	–	227 (33.43)
Rural	–	121 (17.82)
Suburban	–	331 (48.75)
Region of residence		
Northeast	110 (16.15)	110 (16.15)
Midwest	134 (19.68)	134 (19.68)
South	256 (37.59)	256 (37.59)
West	181 (26.58)	181 (26.58)
Survey language		
English	593 (87.08)	593 (87.08)
Spanish	88 (12.92)	88 (12.92)

^a^ Abortion identity was measured with nine response options and collapsed into five categories for analysis: Pro-choice (strongly, moderately, or slightly pro-choice), Pro-life (strongly, moderately, or slightly pro-life), Equally pro-choice and pro-life, Neither pro-choice nor pro-life, and Prefer not to answer

**Table 2:** Primary language proficiency of respondents at Wave 1 (*N* = 681)

	Spanish Survey (*n* = 88) *n* (%)	English Survey (*n* = 593) *n* (%)	Total (*n* = 681) *n* (%)
English Proficient	10 (11.36)	17 (2.87)	27 (3.96)
Bilingual	37 (42.05)	39 (6.58)	76 (11.16)
Spanish Proficient	40 (45.45)	1 (0.17)	41 (6.02)
Non-Hispanic	1 (1.14)	536 (90.39)	537 (78.85)

### Analytical approach

A team of four researchers conducted the analysis; two of them were bilingual and native Spanish speakers, and two of them were English-speakers. Data were analyzed in the original language provided by respondents. Our analytical approach consisted of a nine-step process.

First, the four coders received an initial subsample of responses from 200 participants (i.e., 600 reasons) to be coded independently to generate four preliminary codebooks inductively. The English-speaking coders received the same responses from 200 participants who answered in English, and the bilingual coders received all the responses in Spanish (*n* = 88 participants) and the remaining in English up to 200 participants. This distribution allowed us to ensure that the emerging preliminary codebooks would account for possible differences across languages. Second, the second author reconciled the four individual codebooks into a harmonized codebook, grouped codes into broader thematic categories, and generated definitions for each code. Third, the four coders met to review the proposed harmonized codebook and adjusted it as needed. Fourth, the same two pairs of coders applied the harmonized version of the codebook to responses from a new subsample of 200 participants. Fifth, the second author conducted interrater reliability analysis to assess code application in step four by calculating Cohen’s kappa statistics for both pairs of coders for each code. This analysis identified systematic issues in the application of the codes, with lower Cohen’s kappa coefficients indicating poor agreement among coders. Sixth, the coding team met for a second reconciliation phase of the codebook to assess examples of discrepancies across coders and to discuss the codes in more detail, particularly codes with low inter-rater reliability scores. Discussion of exemplar responses continued until consensus was reached and coders felt a high degree of familiarity with each code and their definitions and applications. All possible disagreements that remained across coders were resolved case-by-case through discussion. Based on this discussion, the codes and corresponding definitions were amended as needed, thus generating a final version of the codebook containing 26 codes (see Table 3).

**Table 3:** Final codebook: themes, individual codes, and definitions

Theme	Code Name	Definition
Unwanted/Unplanned Pregnancy Reasons	Unwanted pregnancy/parenthood	Not wanting/desiring to have a child or be pregnant at the moment or ever (i.e., not wanting to be a parent)
	Unplanned	Lack of birth control, contraception failure or misuse, unplanned/expected pregnancies, mistake
Violence-Related Reasons	Violence-related reasons	Rape, incest by a family member, sexual assault
Health Reasons	Health – general	Unspecified medical or health-related reasons, no specific reference to the mother or fetus
	Health – woman	Physical or mental health of the woman, including substance use or addiction
	Health – fetus	Health of the fetus/baby/unborn, including fetal anomalies, genetic defects, illness, diseases, stillbirths, non-viability
Financial Reasons	Financial reasons	Low income, economic instability, lack of economic resources, inability to afford the cost of having a child or another child, including situations of poverty or lack of housing
Not Ready/Unprepared Reasons	Not ready/unprepared	Not being ready or prepared to parent in general, including not being emotionally ready, or not being mature enough
	Not the right time	Not ready to be a parent/have a child at that particular point in time
	Lack of support/unable to care	Inability to care for or support a child, lack of support from social network to care for a child
Partner-Related Reasons	Man does not want to parent	The man/partner does not want to be a parent
	Unwilling to parent alone	Absence of a man/father and unwillingness to raise a child alone or be a single mother
	Relationship issues	Unstable, negative, abusive, violent, coercive relationship with partner/man/boyfriend/husband
Age-Related Reasons	Age-related reasons	Too young or too old to care for a child
Unacceptable Reasons	Irresponsibility	Judgmental responses about qualifying the woman/couple’s behavior as stupid, nonsense, irresponsible, or illogical
	Misinformation	Judgmental responses about the woman’s agency in the decision-making process
	Selfishness/(in)convenience	Judgmental responses about abortion as selfish, vanity, convenience, inconvenience, immaturity, use as birth control, body image
	Lack of values/immoral behavior	Any other anti-abortion and judgmental responses about morality, lack of values, respect for life, religiosity, abortion considered murder
Personal Reasons	Personal reasons/avoid negative outcome	Non-specified personal reasons and reasons related to avoiding negative outcomes for the child (child suffering), or abortion as a responsible decision
	Personal goals or life plans	Present or future personal goals, education, career, lifestyle, not enough time or lack of time, work-related constraints, including current life situation being incompatible with the responsibility of a child
	Achieved fertility ideals/intentions	Already have a child/children and do not want more
Emotional Reasons	Emotional reasons	Fear, shame, anxiety, social stigma, pressure, social pressure, or family/peer pressure
Other	Blank	Did not enter a response
	Uncodable	Unintelligible responses
	Not enough context	Response is not detailed enough to identify a relevant code
	I don’t know	Responses such as “I am not sure,” “Unsure,” “IDK”

Seventh, the team progressed to the final stage of coding. In this step, 30% of the English responses from the total sample were coded, with 30% overlap between coders using the final codebook, to assess interrater reliability. Cohen’s kappa statistic was applied to the 30% of responses (*n* = 356 participants) coded by all four coders to assess inter-rater reliability for the final coding stage. Results reflected a high degree of agreement, with kappa coefficients greater than 0.80 across all six pairs of coder comparisons (see Table 4 for the list of codes and inter-rater reliability results). Eighth, the remaining 70% of responses were divided into four subsamples, and each subsample was coded independently by one coder. Last, the entire team met to identify broader themes, capturing overarching similarities across our codes. The team conducted descriptive analysis and created visual representations of the coded data to report the findings. We also conducted chi-square tests of association to determine whether the frequency of individual code applications differed among demographic groups, and Cramér’s V was computed as an effect size measure to indicate the strength of the association. When the assumption of adequate expected cell counts was violated (i.e., more than 20% of expected cell counts were less than 5) we conducted Fisher’s exact test instead. We also ran parallel analyses, excluding four low-information codes (i.e., *blank*, *uncodable*, *not enough context*, “*don’t know*”), to examine whether these responses influenced statistical significance.

**Table 4:** Cohen’s Kappa results for inter-rater reliability across coders A, B, C, and D

Theme	#	Code Name	AB	AC	AD	BC	BD	CD
Unwanted/Unplanned Pregnancy Reasons	1	Unwanted pregnancy/parenthood	0.92	0.95	0.93	0.90	0.90	0.97
	2	Unplanned	0.92	0.96	0.96	0.94	0.94	0.98
Violence-Related Reasons	3	Violence-related reasons	0.99	0.99	1.00	1.00	0.99	0.99
Health Reasons	4	Health – general	0.98	0.94	0.94	0.94	0.94	0.96
	5	Health – woman	0.93	0.93	0.95	0.89	0.91	0.93
	6	Health – fetus	0.97	0.97	0.96	0.96	0.95	0.99
Financial Reasons	7	Financial reasons	0.94	0.94	0.95	0.97	0.97	0.97
Not Ready/Unprepared Reasons	8	Not ready/unprepared	0.94	0.93	0.95	0.95	0.93	0.92
	9	Not the right time	0.96	0.93	0.74	0.96	0.78	0.72
	10	Lack of support/unable to care	0.86	0.85	0.86	0.87	0.88	0.85
Partner-Related Reasons	11	Man does not want to parent	0.74	0.79	0.69	0.77	0.66	0.88
	12	Unwilling to parent alone	0.90	0.91	0.89	0.88	0.86	0.94
	13	Relationship issues	0.86	0.84	0.88	0.92	0.80	0.84
Age-Related Reasons	14	Age-related reasons	1.00	1.00	0.99	1.00	0.99	0.99
Unacceptable Reasons	15	Irresponsibility	0.63	0.73	0.81	0.53	0.66	0.69
	16	Misinformation	1.00	0.86	1.00	0.86	1.00	0.86
	17	Selfishness/(in)convenience	0.89	0.83	0.91	0.82	0.94	0.85
	18	Lack of values/immoral behavior	0.82	0.70	0.87	0.62	0.80	0.66
Personal Reasons	19	Personal reasons/avoid negative outcome	0.79	0.75	0.73	0.62	0.70	0.77
	20	Personal goals or life plans	0.63	0.77	0.71	0.75	0.81	0.90
	21	Achieved fertility ideals/intentions	0.63	0.83	0.90	0.70	0.78	0.88
Emotional Reasons	22	Emotional reasons	0.94	0.85	0.89	0.80	0.88	0.76
Other	23	Blank	1.00	0.97	1.00	0.97	1.00	0.97
	24	Uncodable	0.58	0.52	0.66	0.42	0.46	0.66
	25	Not enough context	0.40	0.52	0.48	0.62	0.54	0.49
	26	I don’t know	1.00	1.00	1.00	1.00	1.00	1.00
		Average score	0.85	0.86	0.87	0.83	0.85	0.86

Notes: .90 + Almost perfect; .80-.90 Strong; .60-.79 Moderate; .40-.59 Weak; .21-.39 Minimal; 0-.20 None

### Research team composition and positionality

All team members collaborate on a larger, interdisciplinary project focused on developing survey measures of abortion attitudes in the U.S. from a nonpartisan perspective. This project is guided by advisory input from diverse stakeholders, including individuals and leaders from both the pro-choice/reproductive justice movement and the pro-life/anti-abortion movement. While we aim to approach data collection and analysis with neutrality, we acknowledge that our personal perspectives, lived experiences, and disciplinary training may influence our interpretation of qualitative data when coding and analyzing open-ended responses. We offer this statement to enhance transparency and foster trust in our analytic approach.

## Findings

### Perceptions of the most common reasons people have abortions

In this section, we report descriptive findings of respondents’ perceptions of the most common reasons people get abortions. We first present frequencies from Wave 1 data (681 participants; 2,043 coded reasons) to assess people’s perceptions as a baseline before the *Dobbs* decision. In subsequent sections, we compare frequencies of code application for perceptions after *Dobbs*, as well as between languages.

We identified 10 overarching themes containing the 26 codes present within the data. The prevalence of the themes was the following: (1) *unwanted/unplanned reasons* (17%), (2) *violence-related reasons* (16%), (3) *health reasons* (15%), (4) *financial reasons* (14%), (5) *not ready/unprepared reasons* (11%), (6) *partner-related reasons* (6%), (7) *age-related reasons* (6%), (8) *unacceptable reasons* (6%), (9) *personal reasons* (5%), and (10) *emotional reasons* (5%). Table 5 presents the frequencies of theme and individual code applications. The majority of participants had three codes applied to their provided reasons (i.e., one distinct code for each of their reported three reasons). However, 12.2% of participants had a response with more than one code applied because they mentioned “complex” reasons for abortion. For example, one participant provided as a single reason, “Not ready to care for a child or unplanned pregnancy;” we applied both the ‘not ready/unprepared’ and ‘unplanned’ codes to this response. That some participants’ reasons required more than one code suggest that people’s perceptions of the reasons people get abortions can be complex and interrelated.

**Table 5:** Participants’ perception of the most common reasons for getting an abortion (Wave 1, *N* = 2,043 responses, *N* = 681 respondents)

	Freq	Percent
**Unwanted/Unplanned Pregnancy Reasons**	**349**	**17%**
Unwanted pregnancy/parenthood	206	10%
Unplanned	143	7%
**Violence-Related Reasons**	**330**	**16%**
Violence-related reasons	330	16%
**Health Reasons**	**306**	**15%**
Health – general	122	6%
Health – woman	68	3%
Health – fetus	116	6%
**Financial Reasons**	**279**	**14%**
Financial reasons	279	14%
**Not Ready/Unprepared Reasons**	**223**	**11%**
Not ready/unprepared	91	4%
Not the right time	23	1%
Lack of support/unable to care	109	5%
**Partner-Related Reasons**	**118**	**6%**
Man does not want to parent	23	1%
Unwilling to parent alone	60	3%
Relationship issues	35	2%
**Age-Related Reasons**	**117**	**6%**
Age-related reasons	117	6%
**Unacceptable Reasons**	**123**	**6%**
Irresponsibility	32	2%
Misinformation	17	1%
Selfishness/(in)convenience	44	2%
Lack of values/immoral behavior	30	1%
**Personal Reasons**	**110**	**5%**
Personal reasons/avoid negative outcome	26	1%
Personal goals or life plans	58	3%
Achieved fertility ideals/intentions	26	1%
**Emotional Reasons**	**53**	**3%**
Emotional reasons	53	3%

The most prevalent theme, *unwanted/unplanned pregnancy reasons* (17%), comprised the individual codes ‘unwanted pregnancy/parenthood’ (10%) and ‘unplanned pregnancy’ (7%). ‘Unwanted pregnancy/parenthood’ included responses related to not wanting to parent more generally, such as, “*don’t want kids*,” and “*not wanting children*,” as well as simple statements of, “*unwanted*” and “*unwanted pregnancy*.” The ‘unplanned pregnancy’ code included responses related to lack of family planning (e.g., “*unexpected pregnancy*”), failure to use contraception or misuse of contraception (e.g., “*didn’t use any form of birth control*”), accidental pregnancy (e.g., “*accident*”), being pregnant by mistake (e.g., “*mistake*”), or pregnancy that results from casual sexual relationships (e.g., “*one night stand*”).

The second most prevalent theme was *violence-related reasons* (16%). Some participants provided responses simply stating “*rape*” or “*incest*,” while others added a bit more detail, such as “*did not consent to sex*,” “*sexual assault*,” “*molestation*,” “*sexual violence*,” or “*sexual abuse*.” We note that *violence-related reasons* is the only code that comprises the Violence-related reasons theme and that when comparing frequencies of application amongst single codes (i.e., comparing nonbolded percentages in the rightmost column of Table 5), *violence-related reasons* was the individual code most frequently applied across all responses.

The third most prevalent theme, *health reasons* (15%), comprised the individual codes ‘health – general’ (6%), ‘health – fetus’ (6%), and ‘health – woman’ (3%). ‘Health – general’ included non-specified health circumstances, such as, “*health issue*,” “*medical condition*,” and “*medical necessity*.” ‘Health – fetus’ included references to the “*fetus*,” “*baby*,” “*unborn*,” or “*child*,” and discussed health issues such as “*child would be born with life threatening problems*,” “*abnormalities*,” “*fetal health issues*,” and “*unviable fetus*.” ‘Health – woman’ included references to the pregnant person or mother, such as, “*mother would die if proceeded with pregnancy*,” “*risk of death to mother*,” and “*medical emergency for mother*.” We note that general health issues and the health of the fetus were more frequently cited as perceived reasons for getting an abortion compared with the health of the mother.

The fourth most prevalent theme was *financial reasons* (14%). This theme included reasons such as “*finances*,” “*lack of financial stability*,” “*can’t afford to have a baby*,” “*low income*,” “*poverty*,” and “*not prepared to financially support a child*.” It is worth noting that like *violence-related reasons*, *financial reasons* is both a theme and an individual code, and that when comparing frequencies of application amongst single codes (i.e., comparing nonbolded percentages in the rightmost column of Table 5), *financial reasons* was the second most frequently applied individual code, following *violence-related reasons*.

The fifth most prevalent theme was *not ready/unprepared reasons* (11%). Participants expressed this when discussing the pregnant person being “*not emotionally ready to parent*” or “*not mature enough*,” but also to explain seeking abortion because of “*lack of support or being unable to care*,” or referring to not having adequate support for parenting/continuing with a pregnancy. This theme also includes responses related to the pregnant person feeling unable to care for a child at that moment in time such as, for example, “*it is not the right time*,” “*not now*,” and “*wrong timing*.”

The remaining themes each represent less than 10% of the reasons mentioned by respondents. The theme/code of *age-related reasons* (6%) typically included reasons such as, “*too young*,” “*underage*,” and “*teenage pregnancy*” and to a lesser extent, the person being “*too old*” to have a child. The theme *partner-related reasons* (6%) included individual codes of ‘unwilling to parent alone’ (3%; e.g., “*single*,” “*unattached to father and can not parent alone*”), ‘relationship issues’ such as unstable, violent, or coercive relationships (2%; e.g., “*bad relationship*”), and ‘man does not want to parent’ (1%, e.g., “*person who fathered is denying and does not want to be a father*”). Finally, we identified a set of reasons that we defined as *unacceptable reasons* (6%), with participants expressing critical or judgmental attitudes when defining reasons that reflected a lack of responsibility, knowledge, or morality. This theme comprised the codes: ‘irresponsibility’ (2%; e.g., “*stupidity*”), ‘misinformation’ (1%; e.g., “*lack of education or belief about a preborns life and personhood*”), ‘lack of values’ (1%; e.g., “*they prefer to kill a unique human being*”), and ‘selfishness/(in)convenience’ (2%; e.g., “*self-centered*”), which also included use of abortion as birth control (e.g., “*use abortions as birth control*”).

The final two themes accounted for 5% or fewer of responses. *Personal reasons* (5%) included reasons such as ‘personal goals or life plans’ (3%) including “*career development*,” “*continuing education*,” “*lifestyle preference*,” “*lack of time*,” “*work-related constraints*,” or other “*life situations incompatible with the responsibility of having a child*.” To a lesser extent, this theme also included ‘achieved fertility goals’ (1%; e.g., “*already have all children they want*”) and general ‘personal reasons/beliefs’ (1%; e.g., “*because she wants one*,” “*personal choice*,” “*because they are being a responsible human being and not beinging* [sic] *an unwanted being into the world*”). Finally, *emotional reasons* (3%) included responses such as, “*scared*,” “*they will be vilified by family and community*,” and “*fear of the unknown*.”

### Changes in perceived reasons for getting an abortion after the *Dobbs* decision

**Fig. 1:**
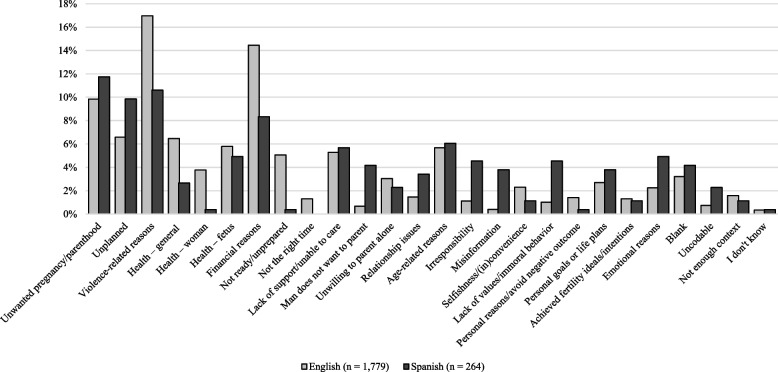
Participants’ changes in perception of the most common reasons for getting an abortion before and after the *Dobbs* decision (Two waves, *N* = 4,086 responses, *N* = 681 respondents)

With the *Dobbs* decision implemented between Wave 1 and Wave 2 of data collection, the longitudinal nature of the data allowed us to observe potential differences across waves (see Fig. 1) and may offer insights into the impact of *Dobbs* on respondents’ perceptions of the most common reasons people get abortions. This analysis included 681 participants (4,086 coded responses) who responded to the open-ended item in both waves. Using all 26 codes, we ran a chi-square test of association and found significant differences in participants’ responses between Wave 1 and Wave 2, χ^2^[25] = 44.46, *p* < 0.01, Cramér’s V = 0.102. We also conducted a separate chi-square test to specifically examine whether the decrease in low-information responses across waves was statistically significant. This analysis confirmed a significant reduction in these response types (*blank*, *uncodable*, *not enough context*, and “*don’t know*”), χ^2^[3] = 13.56, *p* = 0.004, Cramér’s V = 0.240. Thus, when we excluded low-information responses, this difference was no longer statistically significant, χ^2^[21] = 30.12, p = 0.090, Cramér’s V = 0.087. These results suggest that changes in low-information responses accounted for much of the observed shift between waves. For specific reasons, the largest observed difference was a higher frequency of respondents mentioning ‘financial reasons’ (+ 4%), and any of the ‘health reasons’ (+ 3–4%) after the *Dobbs* decision. Alternatively, there was a decrease in the frequency of respondents mentioning ‘lack of support/unable to care’ (−5%), ‘unwanted pregnancy/parenthood’ (−3%), ‘unplanned’ (−2%), ‘not the right time’ (−2%), and ‘unwilling to parent alone’ (−2%), as well as fewer participants leaving one of the boxes blank (−5%) after *Dobbs*.

### Differences in perceived reasons between languages

**Fig. 2:**
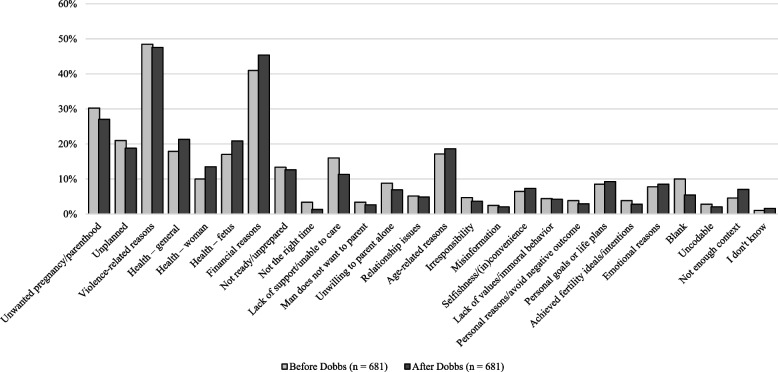
Participants’ perception of the most common reasons for getting an abortion by language (Wave 1, *N* = 2,043 responses, *N* = 681 respondents)

We also explored potential differences in responses by participants’ language. This analysis draws on responses from Wave 1 only, with a total of 2,043 coded responses (1,779 in English and 264 in Spanish). Figure 2 presents differences in the frequency of individual code application by language (English and Spanish). Using all 26 codes, a Fisher’s exact test revealed a significant difference between languages (*p* < 0.001), Cramér’s V = 0.277. This difference remained significant when excluding low-information responses (*p* < 0.001), Cramér’s V = 0.281. English-speaking respondents mentioned ‘violence-related reasons’ (+ 6%), ‘financial reasons’ (+ 6%), and ‘not ready/unprepared’ (+ 5%), more often than Spanish-speaking respondents. Spanish-speaking respondents mentioned ‘unwanted pregnancy/parenthood’ (+ 2%), ‘unplanned’ (+ 3%), ‘man does not want to parent’ (+ 3%), ‘relationship issues’ (+ 2%), ‘irresponsibility’ (+ 3%), misinformation (+ 3%), ‘lack of values/immoral behavior’ (+ 4%), as well as emotional reasons (+ 3%) more often than English-speaking respondents.

To assess whether language-related differences were consistent across time, we conducted additional post hoc analyses (see Table 7 in the Appendix for English- and Spanish-speaking participants’ perceptions before and after *Dobbs*). Using all 26 codes, Fisher’s exact tests revealed significant differences between English- and Spanish-speaking respondents both pre-*Dobbs* (*p* < 0.001, Cramér’s V = 0.277) and post-*Dobbs* (*p* < 0.001, Cramér’s V = 0.239). These differences persisted after excluding low-information codes (pre-*Dobbs*: *p* < 0.001, Cramér’s V = 0.281; post-*Dobbs*: *p* < 0.001, Cramér’s V = 0.231). Within-language analyses showed no significant difference between waves for English speakers (χ^2^[25] = 33.68, *p* = 0.115; 22-code model: χ^2^[21] = 25.53, *p* = 0.225). Among Spanish speakers, a significant difference across waves was found using all codes (Fisher’s exact test: *p* = 0.026), though this was not significant when low-information responses were excluded (*p* = 0.136). These findings suggest that perceptions among English-speaking respondents remained relatively stable across waves, while modest shifts among Spanish-speakers may be driven, at least in part, by changes in the prevalence of low-information responses post-*Dobbs*.

## Discussion

Our research aims in this study were to (1) assess the U.S. public’s perceptions of the most common reasons for someone to seek abortion; (2) determine whether there were differences in perceived reasons before and after the *Dobbs* decision; and (3) identify possible differences by language. The three most prevalent themes of perceived reasons for seeking abortion that we identified were *unwanted/unplanned pregnancy reasons*, abortion sought for *violence-related reasons* such as rape or incest, and the abortion is needed for *health reasons*. Additional perceived reasons, listed in order of prevalence, included *financial reasons*, *not ready/unprepared reasons*, *partner-related reasons*, *age-related reasons*, *unacceptable reasons*, *personal reasons*, and *emotional reasons*. Comparing individual codes, the code for ‘violence-related reasons’ was most frequently applied to responses, followed by ‘financial reasons,’ suggesting that to some extent, perceived reasons for seeking are consistent with reported reasons (e.g., financial reasons), but that people are misinformed regarding other reasons perceived as common when they are not (e.g., violence-related reasons).

### Impact of the *Dobbs* decision on people’s perceived reasons for seeking abortion

After the *Dobbs* decision, we identified an increase in people mentioning *health reasons* and *financial reasons*. Following the *Dobbs* decision, intense media^4^ coverage of changes to abortion legislation and media depictions of abortion following the *Dobbs* announcement [21] may have heightened public awareness of the decision’s implications for abortion access, particularly regarding health-related reasons for seeking abortion [22]. Media can strongly influence public attitudes and knowledge about health and policy issues, such as policing, crime, and sexual permissiveness [23–26] as well as abortion [27–31]. Thus, discourse surrounding abortion laws and healthcare may have heightened public awareness of the consequences of the *Dobbs* decision [32].

Immediately after *Dobbs*, media outlets drew attention to trigger laws that had the potential to impose automatic or nearly immediate restrictions on abortion reminiscent of pre-*Roe v. Wade* times, with some even enforcing total bans that exclude certain health-related reasons to obtain an abortion that often serve as exceptions [3].^5^ In the months after *Dobbs*, reports began circulating in the media of medical care being impacted by newly implemented restrictive abortion laws, with physicians themselves noting changes to standards of reproductive healthcare, such as pregnant patients more frequently experiencing preventable complications [34]. Compounding reporting of abortion laws and practices, entertainment and digital platforms also shape abortion discourse and potentially skew public perceptions. Herold [35] found that television programs in the post-*Dobbs* era increasingly depict abortion plotlines with legal and medical complications, often featuring narratives centered around trauma or exceptions (e.g., rape, health risk), which may contribute to a distorted view of abortion-seeking scenarios as primarily commonplace, despite being rare. Similarly, Acero et al. [36] analyzed YouTube videos on abortion procedures and found that anti-abortion videos were significantly more likely to contain misinformation and score poorly on quality and accuracy measures.

Similarly, the increase in responses related to *financial reasons* after *Dobbs* may be linked to media coverage following the fall of *Roe*, which highlighted the significant impact of the *Dobbs* decision on lower-income communities and vulnerable minorities in abortion-hostile states [40, 41]. These populations often face difficulties traveling out-of-state for abortion care and may be forced into continuing their pregnancy, perpetuating financial distress and poverty [42]. Beyond journalism, politicians, celebrities, podcasters, abortion storytellers, and the general public, have actively engaged in visible acts of support or concern [37–39], contributing to widespread public discourse and advocacy around abortion rights.

### Differences across respondents’ language

We observed differences in perceived reasons for seeking abortion across respondents’ language. Specifically, English-speaking respondents more frequently mentioned *violence-related reasons*, *health reasons*, and *financial reasons* compared with Spanish-speaking respondents. Spanish-speaking respondents more frequently mentioned *unwanted/unplanned pregnancy reasons*, *partner-related reasons*, and *unacceptable reasons* compared with English-speaking respondents. These results seem to suggest that Spanish-speaking respondents were keener to report on arguably subjective-type reasons, whereas English-speaking respondents seemed to provide reasons that align with legislative exceptions. These differences may reflect a combination of structural, cultural, and informational factors, as well as a variety of lived experiences among Spanish-language respondents.

Our Spanish-speaking sample represents a diverse population across nativity, citizenship, and linguistic experience (see Tables 8 and 9 in the Appendix). Among Spanish-language respondents, 89% were not born U.S. citizens, but were naturalized citizens. In contrast, nearly all English-language respondents were born in the United States. This difference indicates that survey language was strongly associated with nativity in our sample, though we acknowledge that language is not a perfect proxy for migrant origin or cultural background.

Prior research has noted distinctive family and fertility patterns among foreign-born Hispanic/Latinx populations in the U.S.—such as younger mean age at first birth and a higher presence of unpartnered parenthood [43]—potentially explaining why Spanish-speaking respondents perceived *unwanted*, *unplanned*, and *partner-related reasons* as common reasons why someone might seek abortion. In addition, previous research on abortion attitudes provides evidence that foreign-born Hispanic/Latinx populations tend to hold more conservative views toward abortion [44, 45], often rooted in moral and religious-based arguments [46, 47]. Since our sample was significantly foreign-born (89% naturalized), this might explain why our Spanish-speaking sample, compared with the English-speaking sample, provided arguably more critical or *unacceptable reasons* for abortion. Further, English-speaking participants may have had more consistent exposure to English-language mainstream media, which may focus more on highlighting exceptional abortion stories as well as reporting on abortion legislation, particularly post-*Dobbs* [21, 35]. However, our data show that Spanish-language respondents were not exclusively monolingual (see Table 2), suggesting likely exposure to both English- and Spanish-language media. While language differences may influence media exposure and content salience, we caution against assuming stark informational divides. More broadly, differences in perceived reasons between languages may reflect a combination of linguistic access, political orientation, religiosity, and experiential context. Spanish-language respondents represent a highly diverse group; thus, our data do not allow for definitive conclusions based on migrant origin or cultural orientation, and we caution against making cultural generalizations based on language alone.

### People’s perceptions versus reported reasons

The most frequently reported perceived reason for seeking abortion was *unwanted/unplanned* pregnancy. This reason included responses that abortion is sought because the pregnancy is not desired or unanticipated, which can represent a broad array of circumstances and encompasses many of the actual reasons people seek abortion. Indeed, although the decision can be complicated by mixed emotions, most abortions are sought in instances of unwanted or unplanned pregnancy, with the minority of abortions being sought for “medically indicated” reasons in the context of wanted and/or planned pregnancies [48]. There are a variety of circumstances that contribute to a pregnancy being undesired, such as lack of support in pregnancy and parenting, being unprepared to parent, and inopportune timing, all reasons which were reflected in participants' perceptions within our sample as well as actual reasons provided by abortion seekers [5]. Because the responses comprising this theme were somewhat broad and at times vague, it is challenging to compare the extent our findings align with pregnant people’s reported reasons for seeking abortion. Perhaps participants were thinking quite broadly about reasons for seeking abortion, or considering the most fundamental reason abortion would be sought—that the pregnancy is unwanted or was not planned. After all, if abortion is being sought, then on some level, the pregnancy is unwanted to some degree, even in situations where people feel they need to discontinue a wanted pregnancy for medical reasons. People may have been contemplating this thought when providing their rationale.

In other ways, our findings reveal a palpable discrepancy between people’s perceptions of reasons for seeking abortion and the reality of abortion decision-making. While respondents in our sample frequently mentioned *violence-related reasons* (i.e., rape or incest) and *health reasons* as the second and third most common themes of reasons people get abortions, these two reasons are, in reality, relatively uncommon. Data collected from U.S. abortion patients in 1987 and 2004 indicated that, in both years, 3–4% of patients reported health problems, either personal or fetal health, and 1% reported rape-related pregnancy as their most important reasons for having an abortion [11]. Similarly, among a sample of 953 women who sought abortion, Biggs et al. [5] found that only a minority of women sought abortion because of health-related reasons that affected the woman (11%) or the fetus (5%). Thus, contrary to what was perceived among our participants, only a small minority of pregnant people seek abortion due to rape-related pregnancy or for health-related reasons.

Notably, in the Biggs et al. [5] study, people reported choosing abortion because of the pregnant person’s health more commonly than because of the fetus’ health. This contrasts with our results, given that our participants mentioned the health of the fetus almost twice as many times as the health of the woman. This might not be surprising given previous research on abortion attitudes in the U.S., which finds that people tend to express unconditional compassion for the *unborn* compared with the pregnant person [49], which may be a result of extensive media coverage that focuses on the fetus rather than the pregnant person [50]. Additionally, health/life endangerment and rape/incest-related reasons are also more palatable to the public as people are far more inclined to endorse legal abortion for these circumstances than others [9, 51, 52]. As such, perhaps our respondents were simply more inclined to provide responses that indicated reasons they were comfortable or more tolerant of people seeking abortion.

Our participants also commonly mentioned *partner-related reasons*, such as parenting without support, as reasons for choosing abortion. Partner-related reasons as motivation for seeking abortion have been commonly reported among abortion patients [5, 11]. For example, Chibber et al. [53] found that a third of women in their study provided a partner-related reason, such as having a poor relationship, a partner’s unwillingness to support a child, or undesirable partner characteristics, which aligns with some of the specificities provided by our respondents.

Respondents sometimes provided reasons for obtaining an abortion that they believed to be *unacceptable*, with implications of judgment of women who have abortions as ‘irresponsible’ or ‘selfish.’ This sentiment stands in contrast with the rationale from women who report their decision to obtain abortions as a form of responsibility. For example, Finer et al. [11] found that women perceived their abortion as a responsible decision when they were unable to provide for a child or when having a child would limit their capacity to properly care for their current children. This disconnect between how people perceive the decisions of others who have abortions and how people who have abortions perceive their own decisions may be on the one hand, a product of people’s judgment of others’ decisions, and on the other hand, a reflection of people themselves justifying making the best decision given the circumstances they are in [54]. In addition, devaluing social norms, legislation, and judicial action related to abortion has the potential to influence attitudes toward abortion and create and reinforce abortion-related stigma, which may explain some people’s articulation of abortion as irresponsible [55–57].

In sum, our findings demonstrate misconceptions regarding beliefs related to pregnant people’s abortion decision-making. Such misconceptions might originate from political and legislative discourse, news media, and other sources of information that tend to highlight abortions due to violence and medical reasons [35, 58–60]—circumstances that are often included as legal “exceptions” in legislation regulating and restricting abortion [61]. Because these types of reasons are more frequently depicted in political debates, laws, and news coverage, in addition to perhaps being more salient, they may be perceived by the public as “good” or “more legitimate” reasons for abortion compared with other reasons people may deem “bad,” or “less legitimate” [62, 63]. Considering that some respondents also perceived that *not ready/unprepared reasons* or *age-related reasons* are common reasons people have abortions, it is likely that people’s views might be influenced by cultural references. Specifically, the entertainment industry (i.e., TV shows, movies, pop culture) often depicts abortion-seekers as scared and vulnerable teenagers who decide to have an abortion without telling their parents [64, 65]. In reality, women who obtain abortions do not fit this depiction [66], as most women seeking abortion are in their 20s or 30s and often have at least one child [67]. And most minors who seek abortion do so with their parents’ knowledge [68]. Given that both traditional and online media are widely accessed sources of health information, these findings suggest that exposure to selective, dramatized, or misleading portrayals of abortion, absent of evidence-based information, may contribute to public misconceptions about *who* seeks abortions and *why*.

### (Mis)Alignment between people’s perceptions and abortion reasons included in surveys

Our findings suggest that people’s perceptions of the most common reasons for obtaining an abortion, while partially distant from reality, do align to a certain extent with abortion circumstances commonly included in national polls and surveys. Abortion due to *violence-related reasons* and as a result of *health reasons* are commonly included in many polls and survey items (for review, see [14]). Indeed, three of the six specific abortion circumstances listed in the GSS—a commonly used national data set to assess abortion attitudes [69]—refer to rape, maternal health, and fetal anomalies. These reasons are also often listed as exceptions in abortion legislation [3, 4]. In other words, these three reasons are *publicly* known, endorsed, and justified, which could explain their endorsement as a common reason for seeking abortion in our sample. Alternatively, our findings suggest other reasons continue to be less known and more stigmatized [62, 63]. More general reasons shared by our participants, such as *unwanted/unplanned pregnancy reasons*, could also align with close-ended survey items that provide the option “if the woman wants it for *any* reason,” which is also fairly vague. This item was introduced in 1977 as a seventh abortion circumstance to the GSS’ abortion legality item (for an overview of this item, see [67]).

Notably, some of the reasons that could potentially be included under the umbrella of *unwanted/unplanned pregnancy reasons* are *partner-related reasons*. These reasons were present among our participants, ranging from the woman not wanting to parent alone, to the man not wanting to have the child, and to various relationship problems faced by the couple. However, the GSS contemplates only a very particular scenario related to *partner-related reasons*, “if the woman does not want to marry the man.” In light of the diverse circumstances that involve the man in an abortion scenario, the GSS item seems to be rather simplistic and outdated [71]. Similarly, only a small proportion of participants in our sample mentioned having met one’s intended number of children as a reason for seeking abortion. Thus, this reason may not be salient within public perception, and the GSS item “if she is married and does not want any more children” seems to reduce the scenario of having met fertility intentions only to married couples.

As calls for more nuanced research and measurement regarding abortion attitudes abound (e.g., [36, 37, 53, 54]), it may be important to consider participant-driven contexts on such circumstance-based survey items rather than primarily researcher-derived contexts like what are included in the GSS [15]. Findings from the present study, potentially cross-referenced with other formative work and actual reasons people seek abortion, could be fruitful in terms of designing novel and important circumstance-based questions for national polls and surveys.

### Limitations

This study entails some limitations. First, our longitudinal study had a short follow-up phase (from June to October 2022), which might have limited the observation period to document meaningful changes between the first and second survey waves. Second, from a cognitive perspective, we need to consider the extent that participants carefully responded to our survey question (i.e., what are the three *most common* reasons for obtaining an abortion). Even though we solicited the most common reasons people seek abortion, we cannot determine with absolute certainty whether respondents just listed reasons for obtaining abortions they knew or had heard about, and potentially did not necessarily think about how often abortion is sought for these particular reasons. Second, even when providing an open-ended response option in a survey, it is often difficult for surveyors to further explore what participants mean when their responses are broad and could encompass a vast array of ideas (e.g., ‘unwanted pregnancy/parenthood’). Additional qualitative work in this regard could be fruitful to provide an opportunity for additional probing. Third, we did not provide participants with a definition of abortion, which may have led to varied interpretations of what constitutes abortion, such as procedural versus medication abortion or self-managed abortion versus abortion under the care of a healthcare provider. These differences in interpretation could have influenced respondents’ perceptions of the most common reasons for seeking abortion. Fourth, the survey did not include a question assessing personal experience with abortion (i.e., whether respondents or someone close to them had ever had an abortion). Future research should explore how personal experience may influence perceptions of abortion. Fifth, our study relied on a binary sex measure (“male” or “female”) provided by Ipsos, which limits our ability to examine responses across diverse gender identities. While we use sex-based terms in our reporting, we recognize this may not reflect all respondents’ gender identities and consider this a limitation of our demographic data. Finally, while our survey used the Ipsos KnowledgePanel®, which is a probability-based panel designed to represent the U.S. household population, panel-based surveys may underrepresent certain groups, such as individuals who are less technologically savvy or less inclined to participate in web-based research and we are not able to weight data derived from open-ended questions. These potential biases should be considered when interpreting the generalizability of our findings.

## Conclusions

We explored English- and Spanish-speaking U.S. adults’ perceptions of the most common reasons people seek abortion through analysis of an open-ended survey item. We also explored differences before and after the *Dobbs* decision, as well as differences in perceptions by language. Participants in our sample perceived that abortions are most commonly sought for *unwanted/unplanned reasons*, *violence-related reasons*, and *health reasons*.

We identified changes in perceptions after the *Dobbs* decision. Specifically, *health* and *financial reasons* were more frequently cited after the decision, suggesting that media coverage and policy changes may have influenced public understanding. Additionally, we found significant differences in perceived reasons by language, which highlights complexities in understanding reasons for seeking abortion. English-language respondents more often cited reasons aligned with legislative “exceptions” (e.g., rape, fetal health), whereas Spanish-language respondents more frequently mentioned subjective or life-contextual reasons (e.g., relationship instability, unwanted pregnancy), reflecting potentially distinct informational, cultural, or experiential frames.

Finally, we identified discrepancies between people’s perceptions of the most common reasons *why* people have abortions (i.e., *unwanted/unplanned pregnancy reasons*, *violence-related reasons*, *health reasons*) and the actual reported reasons *why* people have abortions (i.e., dramatic life changes, financial reasons, and partner-related reasons). Given this, we argue that it is important to ensure that the general public has a better understanding of the motivations behind abortions to correct or prevent further misconceptions in shaping public opinion and policy decisions. Stereotypes and misconceptions have relevant social and policy implications, given that they can create, reinforce, and exacerbate abortion-related stigma, influence public opinion, and shape the policy and legislative landscape on abortion leading to potential misalignment with the lived experiences of abortion-seekers. As some states continue to pass legislation restricting or banning abortion, often naming (rare) exceptions based on specific reasons (i.e., fetal health, woman’s health, rape), it is critical to promote accurate public understanding of abortion motivations. This includes not only correcting misinformation but also expanding the narrative beyond “exceptional” cases. As the legal landscape continues to shift post-*Dobbs*, research efforts should focus on systematically documenting both people’s perceptions and people’s reported reasons for having abortions to ensure alignment of public sentiment and policy post-*Dobbs*.

## Figures and Tables

**Table 6:**
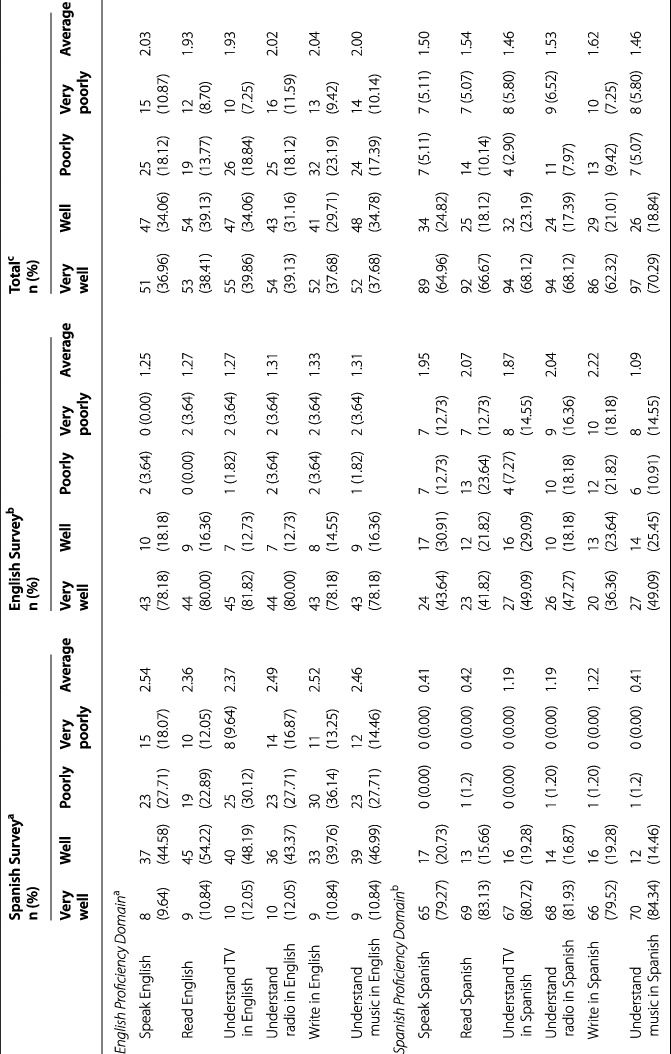
Self-reported English and Spanish language proficiency across six domains for participants who speak a language other than English in their home (*N* = 138)

^a^English proficiency domain: Mean = 2.46, SD = 0.810; Spanish proficiency domain: Mean = 1.19, SD = 0.389

^b^English proficiency domain: Mean = 1.29, SD = 0.641; Spanish proficiency domain: Mean = 2.01, SD = 1.03

^c^English proficiency domain: Mean = 1.99, SD = 0.940; Spanish proficiency domain: Mean = 1.52, SD = 0.819

## Data Availability

Availability of data and material: The data that support the findings of this study may be available from the corresponding author [KNJ], upon reasonable request.

## Appendix

**Table 7:** English- and Spanish-speaking participants’ perception of the most common reasons for getting an abortion before and after *Dobbs* (*N* = 681 respondents)

	Before *Dobbs*			After *Dobbs*		
	English (*n* = 593)	Spanish (*n* = 88)	Difference (English–Spanish)	English (*n* = 593)	Spanish (*n* = 88)	Difference (English–Spanish)
**Unwanted/Unplanned Reasons**						
Unwanted pregnancy/parenthood	30%	35%	-6%	26%	31%	4%
Unplanned	20%	30%	-10%	19%	15%	-5%
**Violence-Related Reasons**						
Violence-related reasons	51%	32%	19%	50%	32%	-18%
**Health Reasons**						
Health – general	19%	8%	11%	23%	9%	-14%
Health – woman	11%	1%	10%	15%	7%	-8%
Health – fetus	17%	15%	3%	22%	11%	-11%
**Financial Reasons**						
Financial reasons	43%	25%	18%	47%	38%	-9%
**Not Ready/Unprepared Reasons**						
Not ready/unprepared	15%	1%	14%	13%	9%	-4%
Not the right time	4%	0%	4%	2%	0%	-2%
Lack of support/unable to care	16%	17%	-1%	11%	10%	-1%
**Partner-Related Reasons**						
Man does not want to parent	2%	13%	-10%	2%	7%	5%
Unwilling to parent alone	9%	7%	2%	7%	6%	-1%
Relationship issues	4%	10%	-6%	4%	8%	4%
**Age-Related Reasons**						
Age-related reasons	17%	18%	-1%	18%	26%	9%
**Critical Reasons**						
Irresponsibility	3%	14%	-10%	3%	7%	4%
Misinformation	1%	11%	-10%	1%	10%	9%
Selfishness/(in)convenience	7%	3%	4%	7%	7%	-1%
Lack of values/immoral behavior	3%	14%	-11%	3%	11%	8%
**Personal Reasons**						
Personal reasons/avoid negative outcome	4%	1%	3%	3%	3%	1%
Personal goals or life plans	8%	11%	-3%	9%	8%	-1%
Achieved fertility ideals/intentions	4%	3%	0%	3%	1%	-2%
**Emotional Reasons**						
Emotional reasons	7%	15%	-8%	7%	20%	14%
**Other**						
Blank	10%	13%	-3%	5%	6%	0%
Uncodable	2%	7%	-5%	2%	5%	3%
Not enough context	5%	3%	1%	6%	16%	10%
I don’t know	1%	1%	0%	1%	3%	2%

^*^ Using all 26 codes, Fisher’s exact tests revealed significant differences between English- and Spanish-speaking respondents both pre-*Dobbs* (*p* < 0.001, Cramér’s V = 0.277) and post-*Dobbs* (*p* < 0.001, Cramér’s V = 0.239). These differences persisted after excluding low-information codes (pre-*Dobbs*: *p* < 0.001, Cramér’s V = 0.281; post-*Dobbs*: *p* < 0.001, Cramér’s V = 0.231)

**Table 8:** United States citizenship by survey language group (*N* = 668)

	Spanish Survey (*n* = 87) *n* (%)	English Survey (*n* = 581) *n* (%)	Total (*n* = 668) *n* (%)
Yes	44 (50.57)	572 (98.45)	616 (92.22)
No	43 (49.43)	9 (1.55)	52 (7.78)

**Table 9:** United States birth or naturalization by survey language group (*N* = 615)

	Spanish Survey (*n* = 44) *n* (%)	English Survey (*n* = 571) *n* (%)	Total (*n* = 615) *n* (%)
Born a U.S. citizen	5 (11.36)	534 (93.52)	539 (87.64)
Naturalized U.S. citizen	39 (88.64)	37 (6.48)	76 (12.36)

## Footnotes

1 For an overview of legislative abortion exceptions, see Felix et al. [3] and Guttmacher Institute [4].

2 Notably, the study did not provide a formal definition of abortion. This was an intentional design choice to allow participants to respond in their own words based on their personal interpretations and perceptions, in alignment with the study’s exploratory aims.

3 Ipsos collected a binary demographic item asking, “Are you…?” with response options “Male” and “Female.” They did not collect additional identity or reproductive health-related information. Ipsos discourages researchers from re-asking their standard demographic items to reduce participant burden, so we relied on this variable in all analyses.

4 By “media,” we refer broadly to traditional media (e.g., newspapers, magazines, radio, television), digital media (e.g., Internet, podcasts), and social media platforms (e.g., Instagram, Twitter/X, TikTok).

5 Currently, of the 21 states with near-total abortion bans and early gestational limits, 6 states have no health exception, 10 states have no rape or incest exception, and 13 states have no fatal fetal anomaly exception. However, all 21 states seem to have life exceptions for the pregnant person as long as there is “reasonable/good faith medical judgment” [33].

## References

[1] Bruce TC, Hutchens K, Cowan SK. The “abortion imaginary”: Shared perceptions and personal representations among everyday Americans. Sci Adv. 2024 Mar;10(9):eadj3135. 10.1126/sciadv.adj3135 PMC10901374 38416827

[2] Watson K. Scarlet A: The Ethics, Law, and Politics of Ordinary Abortion. 1st ed. Oxford, United Kingdom: Oxford University Press; 2018. p. 296.

[3] Felix M, Sobel L, Salganicoff A. A Review of Exceptions in State Abortion Bans: Implications for the Provision of Abortion Services. KFF. 2023. Available from: https://www.kff.org/womens-health-policy/issue-brief/a-review-of-exceptions-in-state-abortions-bans-implications-for-the-provision-of-abortion-services/. [cited 2024 May 23].

[4] Guttmacher Institute. State Bans on Abortion Throughout Pregnancy. 2024. Available from: https://www.guttmacher.org/state-policy/explore/state-policies-abortion-bans. [cited 2024 Apr 12].

[5] Biggs MA, Gould H, Foster DG. Understanding why women seek abortions in the US. BMC Women’s Health. 2013 Dec;13(1). Available from: http://bmcwomenshealth.biomedcentral.com/articles/10.1186/1472-6874-13-29. [cited 2018 Nov 26]. 10.1186/1472-6874-13-29 PMC3729671 23829590

[6] Kirkman M, Rowe H, Hardiman A, Mallett S, Rosenthal D. Reasons women give for abortion: a review of the literature. Archives of Women’s Mental Health. 2009;12(6):365–78. 19517213 10.1007/s00737-009-0084-3

[7] Society of Family Planning. #WeCount. 2025; Available from: https://societyfp.org/research/wecount/

[8] Felix M, Sobel L, Salganicoff A. Addressing Abortion Access through State Ballot Initiatives. KFF. 2024. Available from: https://www.kff.org/womens-health-policy/issue-brief/addressing-abortion-access-through-state-ballot-initiatives/. [cited 2024 Feb 7].

[9] Smith TW, Son J. Trends in Public Attitudes towards Abortion. 2013;50.

[10] Torres A, Forrest JD. Why do women have abortions? Fam Plann Perspect. 1988;20(4):169–76. 3243347

[11] Finer LB, Frohwirth LF, Dauphinee LA, Singh S, Moore AM. Reasons U.S. Women Have Abortions: Quantitative and Qualitative Perspectives. Perspectives on Sexual and Reproductive Health. 2005;37(3):110–8. 10.1363/psrh.37.110.05 16150658

[12] Lantz PM, Michelmore K, Moniz MH, Mmeje O, Axinn WG, Spector-Bagdady K. Abortion Policy in the United States: The New Legal Landscape and Its Threats to Health and Socioeconomic Well-Being. Milbank Q. 2023;101(Suppl 1):283–301. 36960973 10.1111/1468-0009.12614 PMC10126955

[13] Weitz TA, Yanow S. Implications of the Federal Abortion Ban for Women’s Health in the United States. Reprod Health Matters. 2008;16(sup31):99–107. 18772090 10.1016/S0968-8080(08)31374-3

[14] Bowman K, Goldstein S. Attitudes About Abortion: A Comprehensive Review of Polls from the 1970s to Today. American Enterprise Institute; 2021. (AEI Public Opinion Studies).

[15] Jozkowski KN, Bueno X, LaRoche K, Crawford BL, Turner RC, Lo W. Participant-driven salient beliefs regarding abortion: Implications for abortion attitude measurement. Soc Sci Q. 2024;105(2):374–91.

[16] Bankole A, Singh S, Haas T. Reasons why women have induced abortions: Evidence from 27 countries. Int Fam Plan Perspect. 1998;24(3):117–27.

[17] Chae S, Desai S, Crowell M, Sedgh G. Reasons why women have induced abortions: A synthesis of findings from 14 countries. Contraception. 2017;96(4):233–41. 28694165 10.1016/j.contraception.2017.06.014 PMC5957082

[18] Ipsos. KnowledgePanel® Design Summary.. Available from: https://www.ipsos.com/en-us/solutions/public-affairs/knowledgepanel. [cited 2025 Apr 1].

[19] Harkness JA, de Vijver FJRV, Mohler PPh. Cross-Cultural Survey Methods. 1st ed. Hoboken, NJ: Wiley; 2003.

[20] Ipsos. KnowledgePanel® Reward. 2025. Available from: https://www.knpanel.com/participate/rewards.html. [cited 2025 Apr 1].

[21] Blaeser J. How abortion coverage changed in the media, according to the data. Politico. 2023 Dec 27; Available from: https://www.politico.com/news/2023/12/27/politics-rules-abortion-coverage-00131080. [cited 2024 Jun 17].

[22] Jozkowski KN, Bueno X, Turner RC, Crawford BL, Lo WJ. People’s knowledge of and attitudes toward abortion laws before and after the Dobbs v. Jackson decision. Sexual and Reproductive Health Matters. 2023 Dec 31;31(1):2233794. 10.1080/26410397.2023.2233794 PMC10424603 37565622

[23] Intravia J, Wolff KT, Piquero AR. Investigating the Effects of Media Consumption on Attitudes Toward Police Legitimacy. Deviant Behav. 2018;39(8):963–80.

[24] Rosenberger JS, Callanan VJ. The Influence of Media on Penal Attitudes. Crim Justice Rev. 2011;36(4):435–55.

[25] Farrar KM. Sexual Intercourse on Television: Do Safe Sex Messages Matter? J Broadcast Electron Media. 2006;50(4):635–50.

[26] Wakefield MA, Loken B, Hornik RC. Use of mass media campaigns to change health behaviour. Lancet. 2010;376(9748):1261–71. 20933263 10.1016/S0140-6736(10)60809-4 PMC4248563

[27] Herold S, Becker A, Schroeder R, Sisson G. Exposure to Lived Representations of Abortion in Popular Television Program Plotlines on Abortion-Related Knowledge, Attitudes, and Support: An Exploratory Study. Sex Roles. 2024 Jan 20; Available from: 10.1007/s11199-024-01448-3. [cited 2024 Feb 12].

[28] Sisson G, Walter N, Herold S, Brooks JJ. Prime-time abortion on Grey’s Anatomy: What do US viewers learn from fictional portrayals of abortion on television? Perspect Sex Reprod Health. 2021;53(1–2):13–22. 34549534 10.1363/psrh.12183

[29] Brooks JJ, Walter N, Rosenthal EL, Folb KL. Contentious Entertainment: The Role of Character and Narrative Features in Shaping Audience Response to Abortion Storylines. J Health Commun. 2022;27(4):232–40. 35786316 10.1080/10810730.2022.2091064

[30] Mulligan K, Habel P. An Experimental Test of the Effects of Fictional Framing on Attitudes*. Soc Sci Q. 2011;92(1):79–99.

[31] Mena-Meléndez L, Crawford BL, Valdez D, LaRoche KJ, Turner RC, Jozkowski KN. Is news consumption related to abortion attitudes? An exploratory study with a nationally representative sample of US adults. Front Commun. 2024;11(9):1422318.

[32] Treder KM, Amutah-Onukagha N, White KO. Abortion Bans Will Exacerbate Already Severe Racial Inequities in Maternal Mortality. Womens Health Issues. 2023;33(4):328–32. 37301725 10.1016/j.whi.2023.04.007

[33] KFF. KFF. 2024. Policy Tracker: Exceptions to State Abortion Bans and Early Gestational Limits. Available from: https://www.kff.org/womens-health-policy/dashboard/exceptions-in-state-abortion-bans-and-early-gestational-limits/. [cited 2024 Aug 9].

[34] Grossman D, Joffe C, Kaller S, Kimport K, Kinsey E, Lerma K, et al. Care Post-Roe: Documenting Cases of Poor-quality Care Since the Dobbs Decision. [Internet]. University of California, San Francisco: Advancing New Standards in Reproductive Health (ANSIRH); 2023. Available from: https://psnet.ahrq.gov/issue/care-post-roe-documenting-cases-poor-quality-care-dobbs-decision/. [cited 2024 Aug 7].

[35] Herold S. “Women’s Lives Are on the Line, and Our Hands Are Tied”: How Television Is Reckoning With a Post-Dobbs America. Womens Health Issues. 2024;34(6):589–96. 39396895 10.1016/j.whi.2024.09.004

[36] Acero N, Herrero E, Foncham J, McIlvaine J, Kayaalp E, Figueora M, et al. Accuracy, Quality, and Misinformation of YouTube Abortion Procedural Videos: Cross-Sectional Study. J Med Internet Res. 2024;26(1): e50099. 39437380 10.2196/50099 PMC11538871

[37] Mane H, Yue X, Yu W, Doig AC, Wei H, Delcid N, et al. Examination of the Public’s Reaction on Twitter to the Over-Turning of Roe v Wade and Abortion Bans. Healthcare (Basel). 2022;10(12):2390. 36553914 10.3390/healthcare10122390 PMC9777967

[38] Valdez D, Mena-Meléndez L, Crawford BL, Arvind A, Jozkowski KN. Online Social Media Reactions to the Overturn of Roe v. Wade: Public Health Implications and Policy Insights. Sex Res Soc Policy. 2023 Oct 25; Available from: https://link.springer.com/10.1007/s13178-023-00892-2. [cited 2024 Jan 22].

[39] Sisson G, Herold S, Woodruff K. “The stakes are so high”: interviews with progressive journalists reporting on abortion. Contraception. 2017;96(6):395–400. 28844876 10.1016/j.contraception.2017.08.005

[40] Dore K. Supreme Court’s overturning of Roe v. Wade will financially hurt the “most marginalized” women, experts say. CNBC. 2022 Jun 24; Available from: https://www.cnbc.com/2022/06/24/roe-v-wade-decision-expected-to-financially-hurt-marginalized-women.html. [cited 2024 Jun 3].

[41] Suleymanova R. The end of Roe v Wade will hurt poor women most, economists warn. Al Jazeera. 2022 Jun 30; Available from: https://www.aljazeera.com/economy/2022/6/30/the-end-of-roe-v-wade-will-hurt-poor-women-most-economists-warn. [cited 2024 Jun 3].

[42] Forouzan K, Friedrich-Karnik A, Maddow-Zimet I. The High Toll of US Abortion Bans: Nearly One in Five Patients Now Traveling Out of State for Abortion Care | Guttmacher Institute. 2023. Available from: https://www.guttmacher.org/2023/12/high-toll-us-abortion-bans-nearly-one-five-patients-now-traveling-out-state-abortion-care. [cited 2024 May 23].

[43] Guzman L, Wildsmith E, Manlove J, Franzetta K. Unintended Births: Patterns by Race and Ethnicity And Relationship Type. Perspect Sex Reprod Health. 2010;42(3):176–85. 20928956 10.1363/4217610 PMC6436107

[44] Bolks SM, Evans D, Polinard JL, Wrinkle RD. Core Beliefs and Abortion Attitudes: A Look at Latinos. Soc Sci Q. 2000;81(1):253–60. 16856266

[45] Bueno X, Montenegro M, Lo WJ, Valdez D, Crawford BL, Turner RC, et al. Migrant Generations and Abortion Circumstances: Assessing Latinxs’ Abortion Attitudes in the US. Sociol Inq. 2024;94(1):130–48.

[46] Bartkowski JP, Ramos-Wada AI, Ellison CG, Acevedo GA. Faith, Race-Ethnicity, and Public Policy Preferences: Religious Schemas and Abortion Attitudes Among U.S. Latinos. Journal for the Scientific Study of Religion. 2012 Jun;51(2):343–58.

[47] Montenegro MS, Solon M, Valdez D, Crawford BL, Turner RC, Lo WJ, et al. Abortion legality and morality: A preliminary investigation examining the influence of religiosity on abortion attitudes among a sample of US Latinxs. Journal of Religion & Society. 2022;24:1–22.

[48] Foster DG. The Turnaway Study: Ten Years, a Thousand Women, and the Consequences of Having—or Being Denied—an Abortion. Simon and Schuster; 2021. 384 p.

[49] Hadfield JI, Dennis B, Bueno X, Crawford BL, Turner R, Lo WJ, et al. Compassion Narratives in Abortion Attitudes: A Critical Interview Study. The Qualitative Report. Accepted;

[50] Woodruff K. Coverage of Abortion in Select U.S. Newspapers. Women’s Health Issues. 2019;29(1):80–6. 10.1016/j.whi.2018.08.008 PMC6295238 30309695

[51] Jozkowski KN, Crawford BL, Hunt ME. Complexity in attitudes toward abortion access: Results from two studies. Sexuality Research and Social Policy. 2018;15(4):464–82.

[52] Jozkowski KN, Crawford BL, Willis M. Abortion Complexity Scores from 1972 to 2018: A Cross-Sectional Time-Series Analysis Using Data from the General Social Survey. Sex Res Soc Policy. 2021;18(1):13–26.

[53] Chibber KS, Biggs MA, Roberts SCM, Foster DG. The Role of Intimate Partners in Women’s Reasons for Seeking Abortion. Women’s Health Issues. 2014;24(1):e131–8. 24439939 10.1016/j.whi.2013.10.007

[54] Cockrill K, Nack A. “I’m Not That Type of Person”: Managing the Stigma of Having an Abortion. Deviant Behav. 2013;34(12):973–90.

[55] Hanschmidt F, Linde K, Hilbert A, Riedel- Heller SG, Kersting A. Abortion Stigma: A Systematic Review. Perspect Sex Reprod Health. 2016;48(4):169–77. 27037848 10.1363/48e8516

[56] Jozkowski KN, Mena-Meléndez L, Crawford BL, Turner RC. Abortion Stigma: Attitudes Toward Abortion Responsibility, Illegal Abortion, and Perceived Punishments of “Illegal Abortion.” Psychol Women Q. 2023;4:03616843231181350.

[57] Kumar A, Hessini L, Mitchell EMH. Conceptualising abortion stigma. Cult Health Sex. 2009;11(6):625–39. 19437175 10.1080/13691050902842741

[58] Sellers FS, Nirappil F. Confusion post-Roe spurs delays, denials for some lifesaving pregnancy care. Washington Post. 2022 Jul 16; Available from: https://www.washingtonpost.com/health/2022/07/16/abortion-miscarriage-ectopic-pregnancy-care/. [cited 2024 Aug 7].

[59] Andrews M. $80,000 and 5 ER visits: An ectopic pregnancy takes a toll. NPR. 2022 Oct 4; Available from: https://www.npr.org/sections/health-shots/2022/10/04/1126594608/ectopic-pregnancy-expensive-new-york. [cited 2025 Apr 24].

[60] Center for Reproductive Rights. Center for Reproductive Rights. 2025. Recent Case Highlights. Available from: https://reproductiverights.org/our-work/case-highlights/. [cited 2025 Apr 24].

[61] Guttmacher Institute. State Laws and Policies As of March 26, 2025. 2025. State Bans on Abortion Throughout Pregnancy. Available from: https://www.guttmacher.org/state-policy/explore/state-policies-abortion-bans. [cited 2022 Jun 7].

[62] Kimport K, Weitz TA, Freedman L. The stratified legitimacy of abortions. J Health Soc Behav. 2016;57(4):503–16. 27856971 10.1177/0022146516669970

[63] Norris A, Bessett D, Steinberg JR, Kavanaugh ML, De Zordo S, Becker D. Abortion Stigma: A Reconceptualization of Constituents, Causes, and Consequences. Women’s Health Issues. 2011;21(3):S49-54. 21530840 10.1016/j.whi.2011.02.010

[64] Herold S, Sisson G. ‘I could see myself doing something like that’: US women’s engagement with characters who experience abortion, adoption and surrogacy on Little Fires Everywhere. Culture, Health & Sexuality. 2023 Jul 31; Available from: http://www.tandfonline.com/doi/abs/10.1080/13691058.2023.2242436. [cited 2024 Feb 28]. 10.1080/13691058.2023.2242436 37548147

[65] Sisson G, Kimport K. Telling stories about abortion: abortion-related plots in American film and television, 1916–2013. Contraception. 2014;89(5):413–8. 24512938 10.1016/j.contraception.2013.12.015

[66] Bartels M. Scientific American. 2024. Who Gets an Abortion in the U.S.? Perceptions Don’t Match Reality. Available from: https://www.scientificamerican.com/article/who-gets-an-abortion-in-the-u-s-perceptions-dont-match-reality/. [cited 2024 May 8].

[67] Jerman J, Jones RK, Onda T. Characteristics of U.S. Abortion Patients in 2014 and Changes Since 2008. Guttmacher Institute; 2016 p. 29.

[68] Ralph L, Gould H, Baker A, Foster DG. The Role of Parents and Partners in Minors’ Decisions to Have an Abortion and Anticipated Coping After Abortion. J Adolesc Health. 2014;54(4):428–34. 24332398 10.1016/j.jadohealth.2013.09.021

[69] Adamczyk A, Kim C, Dillon L. Examining Public Opinion about Abortion: A Mixed-Methods Systematic Review of Research over the Last 15 Years. Sociol Inq. 2020;90(4):920–54.

[70] Hawbaker A, Turner RC, Mena-Meléndez L, Jozkowski KN. A Mixed-Methods Analysis of the “for any reason” Abortion Attitude Item in the General Social Survey. Under Review.

[71] Cowan SK, Hout M, Perrett S. Updating a Time-Series of Survey Questions: The Case of Abortion Attitudes in the General Social Survey. Sociological Methods & Research. 2022;27:004912412110431.

[72] Hans JD, Kimberly C. Abortion attitudes in context: A multidimensional vignette approach. Soc Sci Res. 2014;48:145–56. 25131281 10.1016/j.ssresearch.2014.06.001

[73] Jelen TG, Wilcox C. Causes and consequences of public attitudes toward abortion: A review and research agenda. Polit Res Q. 2003;56(4):489–500.

